# Supplementary material for: Perturbation by Antimicrobial Bacteria of the Epidermal Bacterial Flora of Rainbow Trout in Flow-Through Aquaculture
Source: Biology (Basel). 2022 Aug 22;11(8):1249. doi: 10.3390/biology11081249 (PMC9405476; doi:10.3390/biology11081249)
Supplement: Supplementary file 1 [file biology-11-01249-s001.zip › supplementary materials rt revision.pdf]

Supplemental Materials

Perturbation by Antimicrobial Bacteria of the Epidermal Bacterial Flora of Rainbow Trout in Flow-through Aquaculture

Hajime Nakatani [1], Naoki Yamada [1], Naoki Hashimoto [2], Fumiyoshi Okazaki [2], Tomoko Arakawa [1], Yutaka Tamaru [2] and Katsutoshi Hori [1,*]

[1] Department of Biomolecular Engineering, Graduate School of Engineering, Nagoya University, Furo-cho, Chikusa, Nagoya 464-8603, Aichi, Japan; nakatanih@chembio.nagoya-u.ac.jp (H.N.); shifon0220@gmail.com (N.Y.); arakawa@lalaproduct.com (T.A.)

[2] Department of Life Sciences, Graduate School of Bioresources, Mie University, 1577 Kurimamachiya, Tsu 514-8507, Mie, Japan; pikachu-1125@yahoo.ne.jp (N.H.); okazaki@bio.mie-u.ac.jp (F.O.); ytamaru@bio.mie-u.ac.jp (Y.T.)

* Correspondence: khori@chembio.nagoya-u.ac.jp; Tel.: +81-52-789-3339

Supplementary tables

Supplementary table S1. Primers used in this study

Name	Sequence	Description	Reference
NGS63F	TCGTCGGCAGCGTCAGATGTGTATAAGAGACAGCAGGCCTAACACATGCAAGTC	16S rDNA V1-V2 primer for NGS	37 *
NGS338R	GTCTCGTGGGCTCGGAGATGTGTATAAGAGACAGGCTGCCTCCCGTAGGAGT	16S rDNA V1-V2 primer for NGS	38 **
27F	AGAGTTTGATYMTGGCTCAG	16S rDNA V1-V3 primer for identification of bacteria	38 **
533R	TTACCGCGGCKGCTGRCAC	16S rDNA V1-V3 primer for identification of bacteria	-

*Marchesi et al. (1998) *Appl Environ Microbiol* 64: 795-799
**M.T. Suzuki and S.J. Giovannoni. *Appl. Environ. Microbiol.*, 62 (1996), pp. 625-630

Supplementary table S2. Proportion of the skin mucus occupying bacteria in the river water.

Year / month	2019			2020							
	Oct.	Nov.	Dec.	Jan.	Feb.	Mar.	Apr.	May.	Jun.	Aug.	Nov.
Aeromonas sp.	0.555	0.053	0.027	0.036	0.058	0.128	0.346	0.304	0.152	0.079	0.017
Acinetobacter sp.	0.007	0.000	0.000	0.000	0.000	0.000	0.000	0.005	0.283	0.000	0.000
Flavobacterium psychrophilum	0.000	0.051	0.021	2.373	5.381	0.047	0.089	0.129	0.065	0.000	0.007
Pseudomonas sp.	0.032	0.030	0.253	0.055	0.030	0.000	0.018	0.030	0.174	0.020	0.005
Vibrio sp.	0.000	0.000	0.000	0.228	0.363	0.599	0.496	0.172	0.196	0.020	0.000
Yersinia ruckeri	0.120	0.059	0.883	0.145	0.222	0.296	0.319	0.109	0.022	0.009	0.002

Each value is expressed as % in the total reads.
Red and blue texts indicate highest and second highest values.
Pink cells indicate the month when the proportion of the species in the mucus was high.

Supplementary table S3. The antimicrobial bacteria isolated from the skin mucus of rainbow trout.

Isolated Month/year /place	Clone name	Antimicrobial activity against bacteria				Identified bacterial species (16S rDNA v1-v3)
		Vibrio anguillarum	*Vibrio ordalli*	*Yersinia rckeri*	*Aeromonas hydrophilla*	
Apr. 2019 Uren Aich JP	N5N1 (KHRT1)	○	○	○	○	*Pseudomonas marginalis/rhodesiae*
	N5N6	○	○			*Pseudomonas marginalis/rhodesiae*
Jun. 2019 Uren Aich JP	N6L1	○	○	○	○	*Pseudomonas koreensis*
	N6L2	○		○	○	*Pseudomonas extremorientalis*
	N6L5	○		○	○	*Pseudomonas extremorientalis*
	N6N1				○	*Pseudomonas gessardii*
	N6N5(KHRT2)	○	○	○	○	*Pseudomonas koreensis*
	N6Z1	○	○	○	○	*Pseudomonas koreensis*
	N6Z3	○		○	○	*Pseudomonas extremorientalis*
	N7Ns4(KHRT5)	○	○	○	○	*Pseudomonas protegens*
Aug. 2019 Uren Aich JP	N8L3	○	○			*Pseudomonas parafulva*
	N9L1	○	○			*Pseudomonas parafulva*
	N9L3	○	○			*Pseudomonas extremorientalis*
	N9N1	○	○			*Pseudomonas baetica*
	N10N2	○	○			*Pseudomonas parafulva*
	N9L1-15	○	○		○	*Pseudomonas koreensis*
	N10L2-15	○	○			*Pseudomonas parafulva*
	N10N1-15	○	○			*Pseudomonas parafulva*
Sep. 2019 Uren Aich JP	LT1	n.d.	○		○	*Pseudomonas jessenii*
	LL1	n.d.	○		○	*Pseudomonas koreensis*
	ST5	n.d.	○			*Lactococcus raffinolactis*
	ST2-15	n.d.	○	○	○	*Lactococcus raffinolactis*
	LN1-15	n.d.			○	*Pseudomonas koreensis*
	LT1-15	n.d.	○		○	*Pseudomonas koreensis*
Oct. 2019 Uren Aich JP	Ox10N4-15	n.d.	○			*Chryseobacterium soldanellicola*
Nov. 2019 Uren Aich JP	M11TS1	n.d.		○		*Pseudomonas koreensis*
	L11TS2	n.d.	○			*Yersinia ruckeri*
	S11NS1	n.d.	○		○	*Pseudomonas fragi*
	M11ZS2	n.d.	○		○	*Acinetobacter movanagherensis ?*
	S11TS2	n.d.	○		○	*Pseudomonas koreensis*
	M11TS2	n.d.	○		○	*Yersinia ruckeri*
Sep. 2019 Uren Aich JP	L11TS1	n.d.	○		○	*Yersinia ruckeri*
	M11N1	n.d.		○	○	*Pseudomonas koreensis*
	M11Z3	n.d.		○		*Pseudomonas koreensis*

Dec. 2019 Uren Aich JP	M12T4	n.d.	○	○	○	*Lactococcus chungangensis*
	S12NS1	n.d.	○		○	*Yersinia ruckeri*
	S12NS2	n.d.	○		○	n.d. *Pseudomonas?*
	S12NS3	n.d.	○		○	n.d.
Jan. 2020 Uren Aich JP	L1T1	n.d.	○		○	*Pseudomonas baetica*
Mar. 2020 Uren Aich JP	N3-2	○	○	○	○	*Pseudomonas baetica*
	N3-5	○	○	○	○	*Pseudomonas baetica*
	N3-8				○	*Pseudomonas baetica*
	N3-9	○			○	*Pseudomonas arsenicoxydans*
	N3-11	○	○	○	○	*Pseudomonas baetica*
	L3-2				○	*Pseudomonas koreensis*
	C3-11	○	○		○	*Pseudomonas extremorientalis*
	C3-6				○	*Pseudomonas aylmerense*
	Z3-4	○	○		○	*Pseudomonas arsenicoxydans*
Feb 2019 Ogaki Gifu JP	N1N1	○	○	○	○	*Pseudomonas sp.*
Feb 2019 Ogaki Gifu JP	N1Z3(KHRT3)	○	○		○	*Pseudomonas protegens*

○: Positive for antimicrobial activity (Clear zoon = 1 cm >) n.d.; Not determined.

Supplementary figures

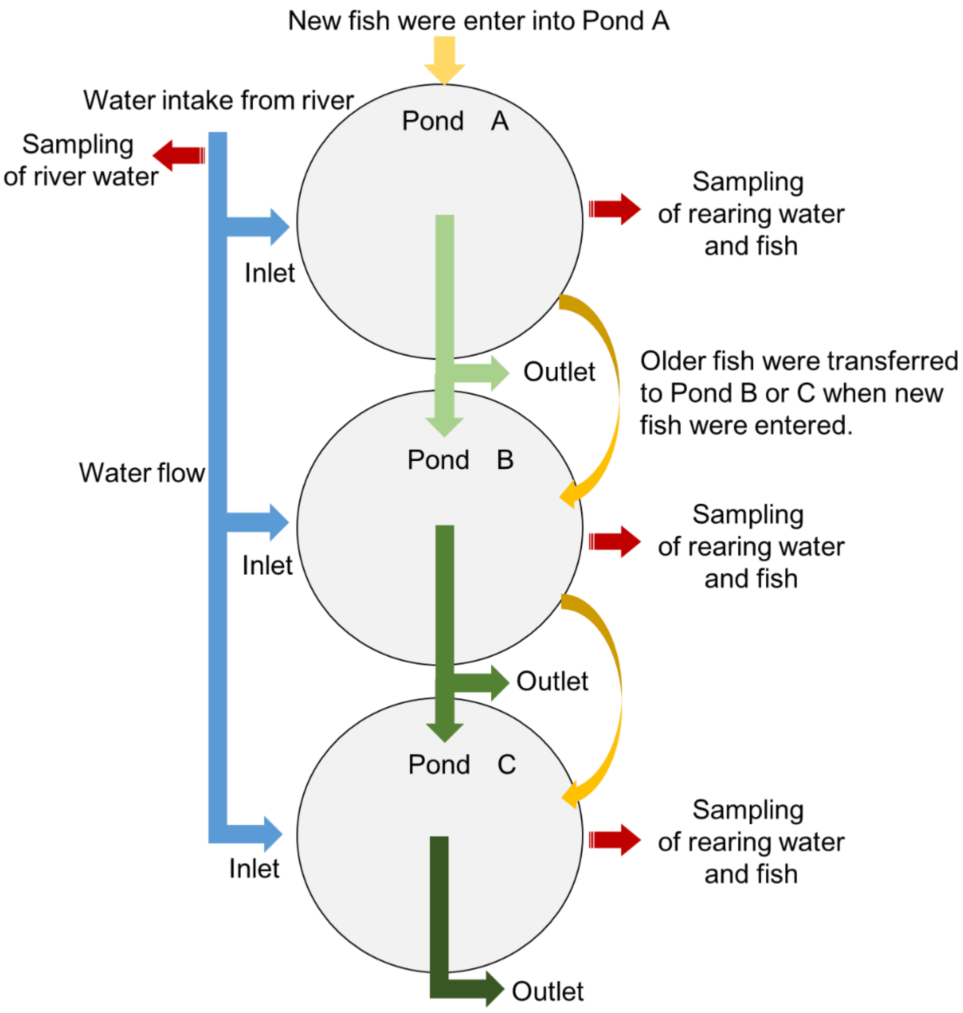

Supplementary figure S1. Schematic figure of the structure of aquaculture ponds and water flow in the ponds. Yellow arrows represent the movement of fish in the system and red arrows represent the sampling positions in the system.

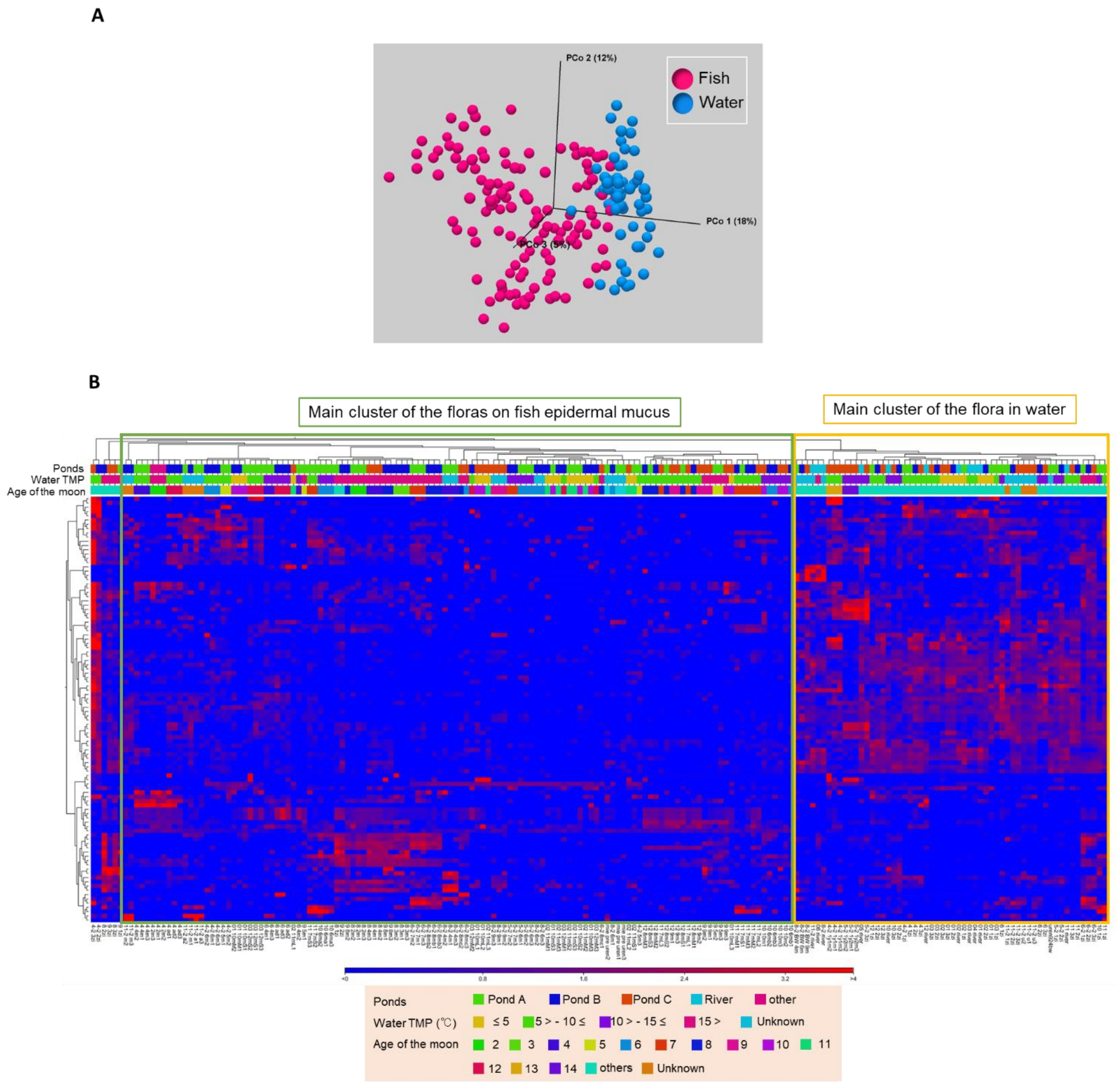

Supplementary figure S2. Beta-diversity of the bacterial floras of rainbow trout skin mucus and water and classification of the bacterial floras by clustered heatmap analysis. (A) The β-diversity using unweighted Unifrac distance was calculated using all the data from the year 2019 and 2020. Data are visualized in a 3D-PCoA plot. (B) The composition of the bacterial flora from mucus samples and rearing water samples was classified based on relative abundance of the top-100 OTUs shown in a clustered heatmap. The green frame represents main clusters formed by the mucus samples, and the yellow frame represents main clusters formed by the water samples. Sampling conditions (aquaculture ponds, water temperature deviation, and age of the moon) are represented by bars with different colors, as defined in the legend.

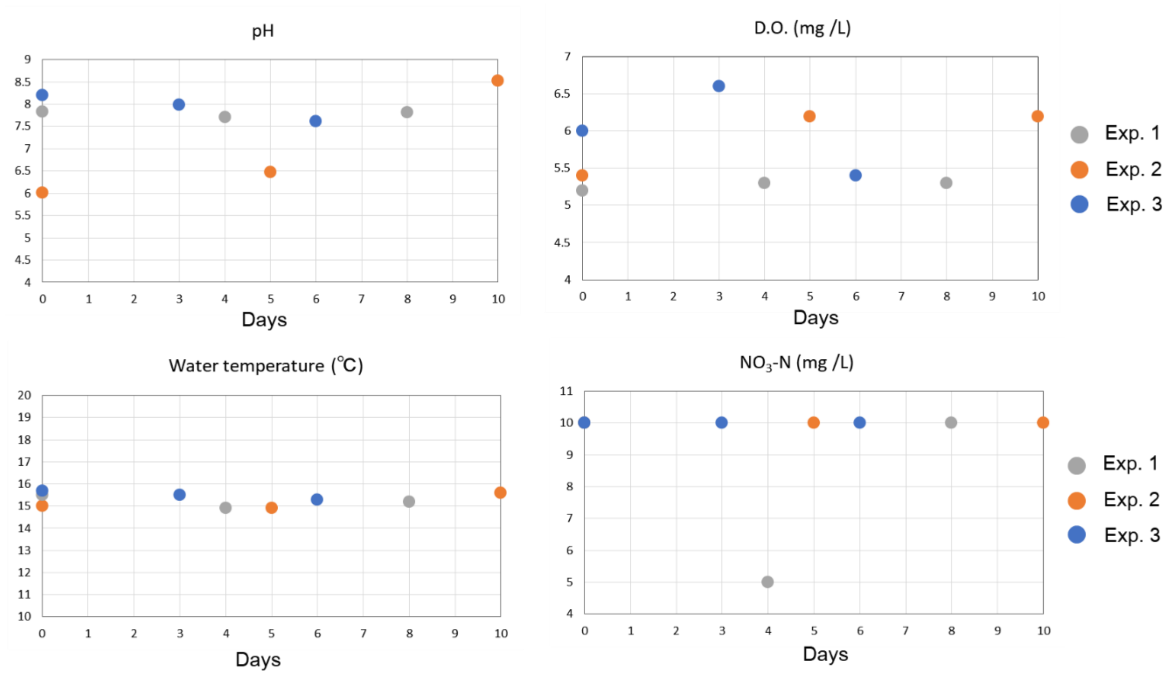

Supplementary figure S3. The changes of the water quality parameters in lab aquarium during the experiments. The pH, dissolved oxygen (D.O.), waterer temperature, and nitrate nitrogen (NO_3-N) were measured at several time points during the experiments.

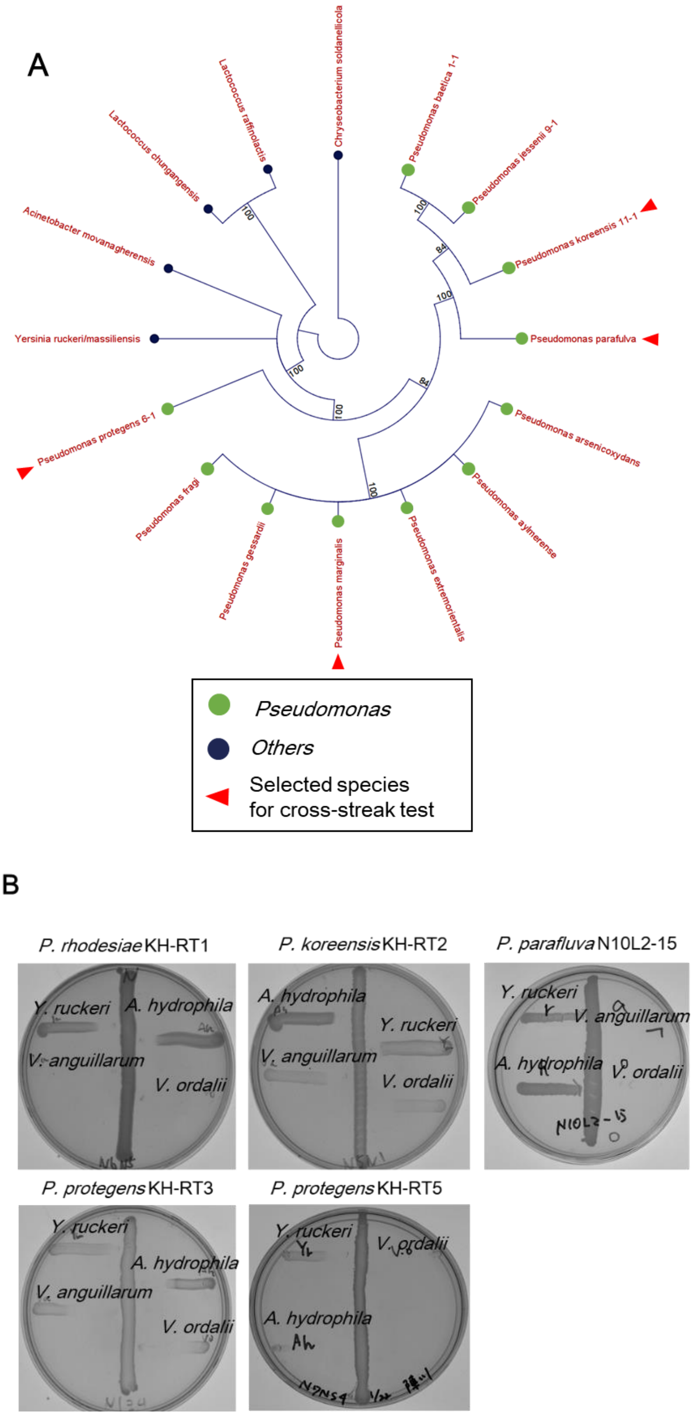

Supplementary figure S4. The phylogenetic analyses the antimicrobial substances-producing bacteria and confirmation of its antimicrobial activity. (A) The phylogenetic analysis was performed with the representative sequence of the 16s rDNA v1-v2 region of antimicrobial substances-producing bacteria. The phylogenetic tree was created by the maximum likely-hood method. The bootstrap values were shown at each branch. (B) The antimicrobial activity of some isolated *Pseudomonas* was confirmed by the cross-streak method.

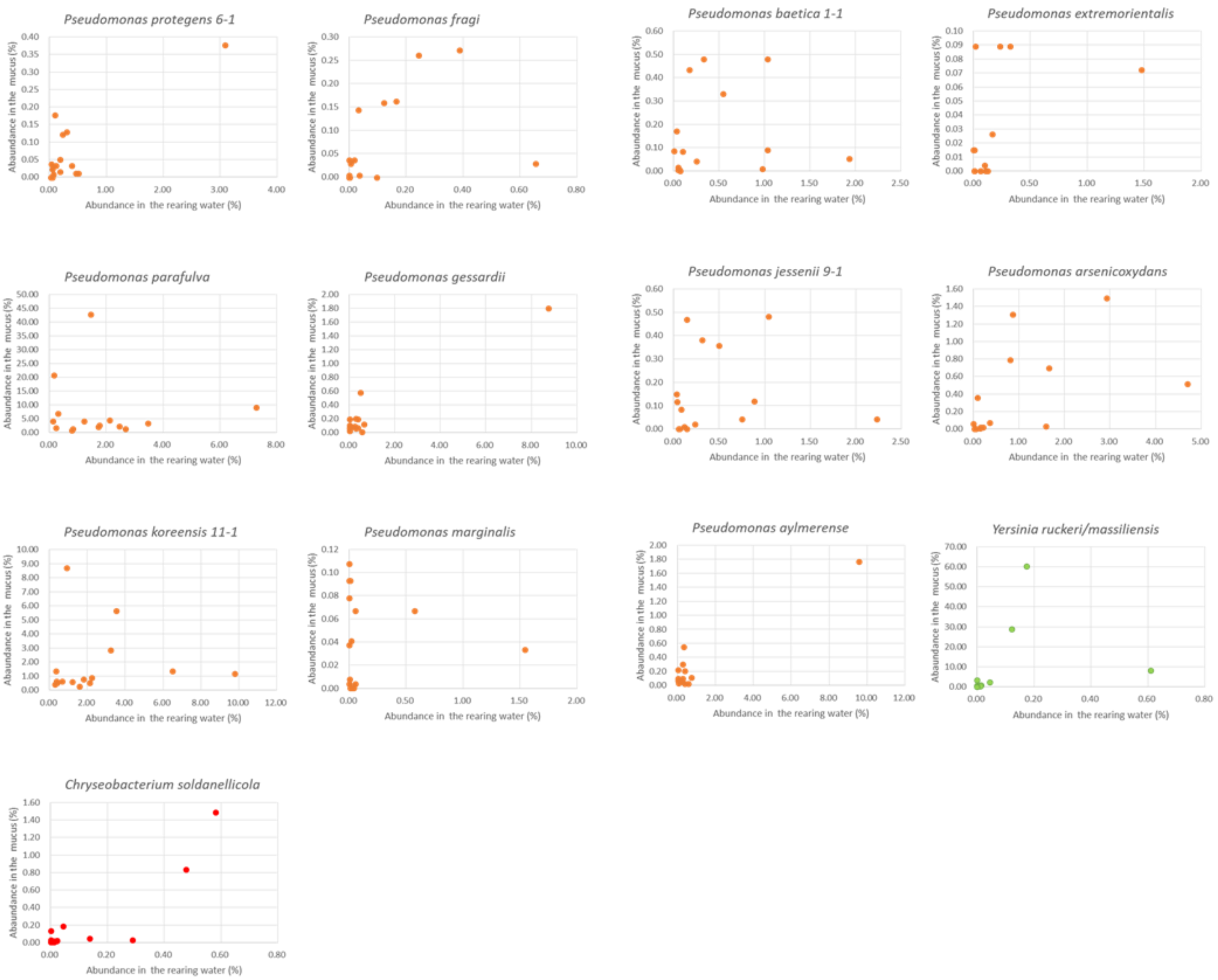

Supplementary figure S5. Correlation diagrams for the abundance of antimicrobial bacteria in the flow-through aquaculture. Correlation between the abundance of the antimicrobial bacteria on the epidermal mucus and that in the rearing water shown in Figure 5 was analyzed. Note that *Lactococcus* and *Acinetobacter* was not analyzed because they were not detected on the mucus or in the rearing water.
